# The New York Institute of Stomatology

**Published:** 1905-07

**Authors:** 

**Affiliations:** The New York Institute of Stomatology


### Reports of Society Meetings.

### THE NEW YORK INSTITUTE OF STOMATOLOGY.

A REGULAR meeting of the Institute was held on Tuesday evening, March 7, 1905, at the Chelsea, No. 222 West Twenty-third Street, New York, the President, Dr. C. O. Kimball, in the chair.

The minutes of the last meeting were read and approved.

### COMMUNICATIONS ON THEORY AND PRACTICE.

*Dr. H. W. Gillett.*—I wish to present the cast of a case showing an extra right superior deciduous lateral. The left deciduous central and lateral are missing, and the permanent central on that side is in place. Please notice the worn facet on the anterior edge of the temporary central, showing where the natural tendency is for the child to bring his jaw to rest as a relief from his usual position of posterior occlusion.

*Dr. W. M. Dailey.*—The case presented by Dr. Gillett is, I think, of unusual importance. I have seen in practice a number of cases of supernumerary deciduous teeth and it has been my opinion for a long time that this condition in almost all cases would cause a malocclusion of the permanent teeth.

It is unfortunate that Dr. Gillett does not show us the lower cast of this case, so that we might see the full results of the malocclusion.

*Dr. F. Milton Smith.*—I wish to again call to the minds of the society the point brought out by Dr. Howland some time ago concerning the use of the tannate of glycerin rubbed on the necks of teeth to reduce sensitiveness. I use it constantly where I have occasion to polish the sensitive necks of teeth, and I have yet to find a case that cannot be relieved in two or three minutes, so that I can do all the polishing necessary.

*The President.*—I may speak of the same thing in my own work. A drop of glycerin in a little tray and some crystals of tannic acid rubbed into it. The sensitiveness stays away. There is no stain.

*Dr. F. L. Fossume.*—I have not had the uniform success with tannate of glycerin that the other two gentlemen have had. Where the teeth have been denuded of the enamel, especially around the necks and in between in the interdental space, and where they are so sensitive that the patient suffers great pain from the slightest irritation, I have found the following treatment to give relief; in fact, I have yet to see the case where it will fail. The rubber dam is to be adjusted on all the teeth to be treated; they are now washed thoroughly with sodium bicarbonate, then dried, and a saturated solution of formalin is applied with cotton pellets and rubbed over the surface with orange-wood sticks until the patient feels no pain. A piece of lintine is to be placed over the patient's nose as a protection against the formaldehyde gas, which is a great irritant to the mucous membrane; in fact, great care must be taken and very small pieces of cotton used. It will also give relief on abraded surfaces on molars. I have used it in very sensitive cavities where the dentine is hard and shiny and where I am sure the pulp is not exposed, my method being to seal it in the cavity for twenty-four hours.

*Dr. Gillett.*—When formalin was first introduced it was advocated for the devitalization of teeth.

*Dr. Fossume.*—I have used it a great deal for this purpose, and I can state positively that it is not a devitalizing agent.

*Dr. E. A. Bogue.*—Years ago there was a mixture put on the market called Naboli. Our friend Dr. Merriam, of Salem, undertook to find out what it contained, and among other things it was found to contain glycerin and tannin. From that a mixture of glycerin, tannin, carbolic acid, alcohol, and atropine was made, and has been used for twenty-five years for sensitive teeth. The glycerin unites directly with the water of the soft part, acting precisely as desiccation would.

*Dr. C. F. Allan.*—The best way to mix glycerin and tannin is in a hot mortar.

*The President.*—I will pass around some radiographs sent in by Dr. Tousey, who expresses his regret that he will not be able to be present to discuss Dr. Schamberg's paper.

Dr. Locherty read a letter from the Connecticut State Dental Society, inviting the members of the Institute to attend their meetings to be held April 18 and 19, 1905.

Dr. Morris I. Schamberg, of Philadelphia, gave an interesting address illustrating his remarks with lantern slides. The following are some of the slides he presented:

Several cases of leprosy showing different stages of the disease. One of facial palsy following involvement of the glands of the subparotid region, due to an abscessed tooth. A number of slides showing syphilitic lesions of the lips and face; also some of epithelioma to illustrate the differential diagnosis. A case of lupus before and during treatment with X-ray. A case of a fractured jaw broken in two places, treated by the Angle method. Radiograph showing appliance in position. Some radiographs of a case of ankylosis of the jaw in which two operations were performed and from one-quarter to one-half inch of bone was removed from each side; the result was satisfactory. A number of radiographs of impacted third molars in various positions. Radiographs of teeth erupting abnormally. Some radiographs showing roots improperly filled, others of abscesses, and several of root amputation.

*Dr. Gillett.*—If I had not seen Dr. Schamberg's root amputation clinic a year ago before the District Society I should possibly not have been so anxious to be here to-night. It was one of the prettiest pieces of work that I have ever seen done. I have been waiting for a case of my own to turn up that I might try it myself. I am a little curious to know where so many cases requiring root amputation come from. It would seem to me that quite a large number of these cases might have been handled in other ways. I would like to know how Dr. Schamberg distinguishes between such cases. It would seem that the simple fact of an abscess of long standing is not a sufficient excuse.

Regarding Dr. Schamberg's work with impacted third molars, I think his statement, that the second molar should never be sacrificed, ought to be widely published. In spite of my decided opinion on this subject, I once had a case where it seemed the only available way. The patient was a chronic invalid, and I did not feel like exposing her to any unnecessary strain. In this case, after the removal of the abscessed second molar I was enabled to clean up the third molar and retain it as the posterior support for a bridge for which the first molar was the main support, using a crown cemented on the first molar with a dowel-pin in a filling in the third molar, according to the method explained by Dr. S. S. Stowell.

It would seem, in extracting a single rooted molar, advisable before removing the crown, as mentioned by Dr. Schamberg, to grasp the whole tooth in the forceps and give it a rotating motion, thus breaking its attachment to the process. This makes the removal of the root easier. I have also found it helpful to split such teeth, taking away, first, the top part, and then lifting out the remainder.

Had I known the course the paper would have taken in relation to impacted wisdom-teeth, I should have been glad to have brought some casts of such a case where I desired to save the patient as much physical suffering as possible. For nearly a year I tried, by means of packing gutta-percha between the second and third molars, to lift the latter tooth up so that I would be enabled to get at it. The results I attained in this way were not very satisfactory. Finally, Dr. Bogue told me of the grass-line wedges, and I set to work with them. In a couple of months I had lifted the molar upward and backward enough so that it could be removed without interfering with the second molar. Grass line is kept by the sporting-goods stores. I found at one store a remnant of a large size that they do not carry in stock generally. They will order any size required.

*Dr. Rice.*—In Dr. Schamberg's picture of impacted wisdom-teeth I noticed none with the molar tipping forward. They were all slanting away from the second molar. I have recently had experience with a case where the third molar was tilted forward, and, indeed, was actually under the second molar. A general surgeon was to remove the tooth, and I was called and asked to furnish appliances in the way of a dental engine, etc. After the patient had been etherized the surgeon decided that he did not know much about it, and asked me to go ahead and take the tooth out. It was a very embarrassing case. There was considerable swelling, and it was very difficult of access. I attempted to use a right-angle bur, but it was not long enough. With a straight bur I attempted to remove the bone sufficiently to take out the tooth. I made a cross-section, and part of the tooth was extracted. At first there was entire relief, but after a few days there was a return of great pain, which was relieved by packing antikamnia into the socket. I would like to know of a practical method of removing a tooth like this without first removing the second molar.

*Dr. E. A. Bogue.*—Referring to what Dr. Gillett has said regarding impacted wisdom-teeth, sometimes these impacted teeth can be saved by wedging them backward and causing them gradually to assume an upright position. Sometimes they can be wedged so as to facilitate their extraction. I have in mind such a case. Dr. Hasbrouck had refused to attempt the operation. I was enabled, by means of tape, to wedge the tooth into a sufficiently upright position to be easily taken out. In many cases that I have seen two or three weeks of preliminary wedging would greatly facilitate the extraction of an impacted tooth, without injury to its neighbors.

Dr. Schamberg has shown us a number of cases of root amputation, and the question came up as to the need of such an operation. I think such operations are very important and necessary many times. I have had such a tooth in my own mouth,—a molar that was doing work for ten or twelve years with only the buccal roots. In one case I amputated the apices of central and lateral incisors, and the cuspid and first bicuspid roots. All these teeth were dead and in bad condition, due to an abscess beginning with the cuspid and spreading to the adjoining teeth. The patient had suffered for thirteen years, and was radically cured of a stubborn neuralgia, as he called it, by the operation.

Dr. Schamberg spoke of a splint used by him in a case of fracture of the lower maxilla. I was disappointed that he did not speak of the interdental splint of rubber, because all of this wiring of the teeth together is injurious and I do not believe that it is often necessary. The dentist, knowing the proper occlusion of the teeth, can take an impression, and then, the lower model being broken apart and the teeth replaced in their proper position relative to the upper teeth, an interdental splint can be made on these models that will hold the mandible in position. Dr. Schamberg will forgive me for reminding him of this omission.

I am much pleased at Dr. Schamberg bringing so prominently before us the very great advantages of the X-ray.

*Dr. Gillett.*—I have seen one case where the third molar was directly over the second molar and the two crowns pointing in exactly opposite directions.

*Dr. F. Milton Smith.*—I have been exceedingly interested in Dr. Schamberg's talk and the pictures he has shown us. He has greatly impressed us with the benefits of the X-ray in our work. The thought given expression to by Dr. Gillett also impressed me, as to the apparent great number of cases in Dr. Schamberg's practice requiring root amputation. I presume it can be partially accounted for from the fact that he has sent him patients from other dentists. In my own practice I have, in five cases found it necessary to remove the end of an abscessed root. I have found exceedingly few cases that I cannot cure.

*Dr. Fossume.*—Relative to the extraction of teeth, I wish to make a plea for greater precaution against infection during the operation and also in the after treatment, and if one is called upon to treat such infected sockets, I know of no more reliable and efficacious germicide than permanganate of potassium. Men making a specialty of the extraction of teeth should at least advise the patient to return to their family dentist to have the dressings removed and the wounds looked after. I have had some difficulty in this direction, and can see no excuse for such neglect.

*Dr. Gillett.*—I would add, "not only after-care, but preliminary care of the mouth before extraction."

I would like to ask Dr. Schamberg if, in amputating the roots of the lower bicuspids and molars, he has any difficulty in avoiding the inferior dental nerve.

*Dr. Schamberg.*—Regarding the apparent frequency with which I perform the operation of root amputation, I admit that I do perform it with more frequency than most practitioners. I will admit that perhaps in the majority of cases of an abscessed tooth, proper medication and treatment will bring about a complete cure. However, there is a large proportion of cases where this method will only bring about a temporary cure, so to speak, but where, owing to the diseased end of the root and surrounding tissue, it is impossible to effect a complete and permanent cure. In these cases surgical interference is desirable and necessary. For that reason I believe that there are many cases where the operation is indicated, but where it is not performed at the present time. There are many persons going around with gum-boils in their mouths who have been told that they are nothing to bother about. There are many roots that it is practically impossible to fill to the very end, and I always make due allowance for root-canal work. We often feel confident that we have reached the end of the root with our filling, when as a matter of fact the X-ray will show that we have not.

Regarding the root of the wisdom-tooth which pointed in an opposite direction from those I showed on the screen, no matter how difficult, the root should be removed, as the prognosis is always bad when a root of this kind is left. Of course, sometimes it is a difficult thing to get the mouth open wide enough. Oftentimes it is absolutely necessary to allow the acute inflammation to subside. The socket of a tooth so extracted should always be packed so that it will heal from the bottom up. I use iodoform gauze for this purpose.

I do not find much danger in interfering with the inferior dental nerve, and even if the nerve is severed it will always unite again.

I do not favor interdental splints, as a rule, because they are not applicable to cases where the fracture is far back toward the ramus. If the mouth is opened in these cases the portion of the fracture at the ramus will not assume its proper relation to the body of the inferior maxilla. I see that the case is kept in a clean condition by having the patient come to me every day for an antiseptic spray between the teeth. The nourishment is also taken in like manner between the teeth.

I do not believe that any operation of the mouth, causing the shedding of blood, should be done without first rendering the mouth as far as possible aseptic. I thoroughly approve of the preliminary use of the permanganate of potash, and I follow up the extraction with an antiseptic wash for some time. The patient should always be impressed with the necessity for treatment of the socket. There should be no deception regarding the failure to remove all the roots. It does not hurt the operator's reputation and it may save the patient's health. Of course, the question of extraction becomes more difficult every day, because we do not now take out teeth that are little decayed, but only complicated cases of impaction come to us for extraction.

I thank you, gentlemen, for your kind attention.

Upon motion, a vote of thanks from the society was extended to Dr. Schamberg for his very interesting and valuable talk.

Adjourned.

Fred. L. Bogue, M.D., D.D.S.,
*Editor The New York Institute of Stomatology.*

